# Status and determinants of enrollment and dropout of health insurance in Nepal: an explorative study

**DOI:** 10.1186/s12962-020-00227-7

**Published:** 2020-10-01

**Authors:** Chhabi Lal Ranabhat, Radha Subedi, Sujeet Karn

**Affiliations:** 1 Policy Research Institute, Sanogaucharan, Kathmandu, Nepal; 2 Manmohan Memorial Institute of Health Sciences, Solteemod, Kathmandu, Nepal; 3 Global Center for Research and Development, Kathmandu, Nepal

**Keywords:** Social health insurance, Dropout, Enrollment assistant, Enrollment, Health care providers, Health insurance board and Nepal

## Abstract

**Background:**

Compared to other countries in the South Asia Nepal has seen a slow progress in the coverage of health insurance. Despite of a long history of the introduction of health insurance (HI) and a high priority of the government of Nepal it has not been able to push rapidly its social health insurance to its majority of the population. There are many challenges while to achieve universal health insurance in Nepal ranging from existing policy paralysis to program operation. This study aims to identify the enrollment and dropout rates of health insurance and its determinants in selected districts of Nepal.

**Methods:**

The study was conducted while using a mixed method including both quantitative and qualitative approaches. Numerical data related to enrollment and dropout rates were taken from Health Insurance Board (HIB) of Nepal. For the qualitative data, three districts, Bardiya, Chitwan, and Gorkha of Nepal were selected purposively. Enrollment assistants (EA) of social health insurance program were taken as the participants of study. Focus group discussions (FGD) were arranged with the selected EAs using specific guidelines along with unstructured questions. The results from numerical data and focus group discussions are synthesized and presented accordingly.

**Results:**

The findings of the study suggested variation in enrollment and dropout of health insurance in the districts. Enrollment coverage was 13,545 (1%), 249,104 (5%), 1,159,477 (9%) and 1,676,505 (11%) from 2016 to 2019 among total population and dropout rates were 9121(67%), 110,885 (44%) and 444,967 (38%) among total enrollment from 2016 to 2018 respectively. Of total coverage, more than one-third proportion was subsidy enrollment—free enrollment for vulnerable groups. The population characteristics of unwilling and dropout in social health insurance came from relatively well-off families, government employees, businessman, migrants’ people, some local political leaders as well as the poor class families. The major determinants of poor enrollment and dropout were mainly due to unavailability of enough drugs, unfriendly behavior of health workers, and indifferent behavior of the care personnel to the insured patients in health care facilities and prefer to take health service in private clinic for their own benefits. The long maturation time to activate health service, limited health package and lack of copayment in different types of health care were the factors related to inefficient program and policy implementation.

**Conclusion:**

There is a high proportion of dropout and subsidy enrollment, the key challenge for sustainability of health insurance program in Nepal. Revisiting of existing HI policy on health care packages, more choices on copayment, capacity building of enrollment assistants and better coordination between health insurance board and health care facilities can increase the enrollment and minimize the dropout.

## Introduction

Promoting well-being by ensuring healthy lives remains one of the key agendas in the Sustainable Development Goals (SDG) adopted by the UN member states in 2015. The main targets on this front is to achieve universal health coverage, including financial risk protection, access to quality essential health-care services and safe, effective and affordable essential medicines and vaccines for all. Provision of health insurance especially for the poor and vulnerable can play an integral role in that direction. At present, very few countries in the world have fully covered health insurance schemes. This is mainly limited to European countries, Japan, South Korea, Australia and Israel having already achieved universal (cent percent) health insurance. Further, countries like Algeria, Mexico, Chile, Slovakia and Turkey have achieved a coverage of more than 90% [1]. At the same time, health insurance systems in some of the developed and particularly developing countries like Nepal also vary in a wide array of dimensions, including risk bearing, choices allowed, sources of revenue and its redistribution, cost saving strategies and presence of specialized and secondary insurance [2].

Evidences from developing countries show that most of them are struggling to expand the coverage of health insurance and there is no sufficient data regarding health insurance coverage at national level. Particularly, in the case of South Asia, the provision of health insurance is at its infancy stage with most countries limited to subsidizing treatment for poor people [3]. Almost, treatment package appears limited to essential health care services and distribution of free medicine are not available throughout the year [4]. There are some state funded pro-poor schemes in India, Pakistan, and Bangladesh focused on primary health care services and some subsidy in maternal and child health, but there is no organized practice considering wide population coverage of health insurance [5]. This has led to increasing commercialization and privatization of health care, creates the ground of expensive treatment and reduces the credibility of public health care facilities [5, 6]. In Nepal, only 5% of population has been covered by HI [7]. Nepal government has prioritized the expansion of HI program however, there are many challenges regarding modality, sustainability, cost effectiveness and quality of health care.

### Theoretical groundings

Globally, there are different philosophical and theoretical discourses regarding health insurance. Scholars are divided on describing health insurance in terms of political ideology or models of political economy and implementation framework. Fundamentally, it is the philosophy of health equity, influenced by political ideology and implemented under the umbrella of social security principles. A majority of schemes provide assurance of need based health care that is mostly based on an individual’s economic capacity and financial contribution [8]. Hoffman [9] describes health insurance in three perspectives: (1) *Health Promotion theory*–relies on using health insurance to pay for medical care that most cost-effectively preserves and improves health; (2) *Financial Security theory*–demands that health insurance limits financial insecurity from these costs and (3) *Brute Luck theory*–highly sensitive to the possibility of adverse-incentive effects arising from moral hazard [9]. Social health insurance is a concept that can play an instrumental role in enhancing social protection and equity. A health insurance system is a means through which healthy people share the risks with unhealthy; young people contribute for the treatment of elderly and children; employed people cover the healthcare costs of unemployed, able people for disable ones etc. [10]. A state operated social health insurance program might ensure that health service is equitable and available to everyone in which the financial contribution is based on income and health service on need [11] since health insurance is social protection of health (SPH). Social protection consists of a menu of policies that addresses health, poverty and vulnerability through user fee removal, fee waivers, and social assistance in healthcare. Social health insurance and other similar schemes such as result-based financing mechanisms are aimed at increasing access to healthcare among disenfranchised communities [12–16]. It has been part of the World Health Assembly agenda and United Nations (UN) General Assembly resolutions [17] and is strongly advocated to become one of the post-2015 millennium development goals namely sustainable development goal (SDG) [18].

In Nepal, there is no defined philosophy and theory regarding current practice of health insurance program. A review of present schemes in operation suggests an amalgam of different hybrid theory and practice in operation, particularly focused towards government’s commitment to UN SDG and upgrading of social health protection. SPH is a simple interaction between the consumer and the provider of healthcare services, in which demand for healthcare services are met by the provision of the service. Consumers are linked to demand side factors (pattern of usage and demand of the population, and the resulting potential workload) while providers are linked to supply side factor (human, physical, and other resources required to provide services). The literature highlights basically four supply side factors that constraint SPH namely, institutions, human resources, infrastructure, and funding [19–23]. These factors are mostly responsible for increasing enrollment and reducing dropout of health insurance with quality health care and determine the overall success of a health insurance program.

For the universal health coverage, financial coverage is the most important part and it is possible if there is > 90% coverage of prepayment health insurance. Under the current health insurance policy, a family of 5 members must pay 3500 Nepalese rupees per year to cover all types of health service with maximum limit of 100,000 rupees [7]. If there are more than 5 members in family, they need to pay 700 rupees per person. Service is provided by registered health care facilities, enrollment assistants at the field enroll in health insurance program, and enrollment officer verifies and approves the intake of a policy. After enrollment, it takes 3-months time to be matured. Insured gets identification card and utilizes the health service. Although, the health insurance program has been expanded in Nepal to about 50 districts, the national coverage (family or individual) is very low. On the other hand, the national drop-out rate of health insurance is increasing in number which is a challenge for the success of this program. Due to the poor intake of health insurance, it is difficult to achieve more than 90% for UHC till 2030. Every year, Nepal government has increased budget for health insurance, but the coverage is not satisfactory and has raised a big question about the sustainability of program itself. In Nepal, currently ‘out of pocket’ payment (OPP) is more than 2/3rd, catastrophic health expenditure at 40% threshold is > 1% and overall UHC index is 46 [24]. This coverage status is mostly from government subsidy groups mainly poor and marginalized people. It has increased financial responsibility of government and a big question on program sustainability. Many private business companies like commercial banks have declared the insurance for their clients but those schemes are not under social security umbrella and under the regulation of health insurance policy.

There is a scant literature on health insurance in the case of Nepal. The literature available basically focus on the enrollment among the poor and health care package/scheme [25], challenges of health insurance [26, 27], etc. Benefit packages and tedious enrollment process were the discouraging factors for enrollment in Community Based Health Insurance (CBHI) [25]. On demand side, socio demographic factors like education, economic status, access to health care facilities etc. are seen responsible for health care utilization in health insurance, but these studies are in small scale [28–31]. A study conducted by Nepal Health Research Council (NHRC) suggests that insured > 90% want to renew the health insurance scheme if it increases the coverage of the service package. About 36% faced difficulties in accessing health care facilities and not satisfied with health workers, and about 40% utilized health service out of scheme [32]. Initially, people who were educated found interested to enroll in health insurance and also, those who belonged to high economic status, access with media and susceptible to disease had better enrollment ratio [33, 34]. Normally, health care utilization is positively associated with health insurance coverage elsewhere [35] and similar trend is found in Nepal as well [31]. Nonetheless, these studies have not explored the factors that might contribute to enrollment from purchaser side. Usually, these studies are lacking a balance in both demand and supply, poor in policy and program review [36] and have drawn one sided conclusion. None of the studies clearly investigated the characteristics of dropout, multiple factors associated with enrollment and drop-out in wide array and how the program would be successful.

This study explores the dropout and enrollment rate of HI program in Nepal and explores the barriers in the field during program operation. Study participants are carefully selected who had a say while observing both demand and supply side and the program implementation. More importantly, this study provides the composite results (demand, supply and policy and program operation perspective) regarding the determinants of enrollment and dropout of HI. The findings mitigate one sided conclusion and argue for practical remedies to reform the existing HI policy, program and implementation bottlenecks.

## Methods

This study implies a mixed method approach to collect data. This includes combining both secondary numerical data from HIB and primary qualitative narratives from the field:

1. Data review: Enrollment and drop out status was calculated based on the numerical data available from the health insurance board of Nepal. The data were clustered by year, district, and enrollment and the drop-out were mapped accordingly. The data were taken from 2016 to 2019 as program piloted and extended and presented by the districts and year with total population, enrollment, subsidy enrollment and dropout. The enrollment is valid for 1 year and dropout is applicable to present next fiscal year and are presented accordingly.
2. Qualitative approach: A field based qualitative study was carried out with the participants and they were well familiar with enrolled individuals and households. The factors causing dropout of health insurance were explored through Focused Group Discussions (FGDs).
  (I) Participants and selection approach: The Enrollment Assistants (EAs) were the participants of focus group discussion. They were primarily responsible for enrollment in health insurance program; and they also had a role to renew the yearly package. In both the roles, they could identify the factors associated with enrollment and drop-out of health insurance in their community, based on their experience and records available. In other words, they had experiences regarding the determinants of enrollment and dropout from demand–supply and program operation side. The participants for FGDs were selected based on the recommendations of the enrollment officers while considering age, sex, education and level of activeness.
  (II) Study sites and justification: Three districts were selected purposively to represent three topographical regions. Gorkha as mountain and hill district, Chitwan as district of diversity and Bardiya as plain district. In each district, we conducted two focus group discussions with total of six focus group discussions. The number of participants in each group was minimum 9 and maximum 11.
  (III) Inclusion and exclusion criteria: Participants who were working as EA in the last 1 year and currently working were included in the FGDs.
  (IV) Study/research tools: For the data collection in study area the following study tools were used.
    - Focus group discussion guideline including key questions: Open ended questions were prepared focusing on characteristics of dropout enrollee, determinants of enrollment and dropout of health insurance and some probing questions to operate FGD.
    - Individual note and diary: To capture thickness in qualitative data, diary and notes were maintained. Further, the cases were recorded with specific incidence to reduce information bias. Four types of codes were used to themetise the note. Code 01 was general characteristic who were unwilling to enroll and dropout insuree. Code 02 factors responsible on enrollment and dropout. It was divided into code 02-1, factors related to health service delivery, 02-2, factors associated to program operation by health insurance board and 02-3, factors related to program and policy. Code 03 was related to special examples or events that expressed by participants in their own language. District and group code were identified with first letter of district (B Bardiya, C, Chitwan and G Gorkha), group code as G1 and G2. For example B-G2-02-01 states for Bardiya district group 2 and factors related to health service delivery.
    - Recorder: Participants voices were recorded in smart phone and some video clips were also recorded.
3. Results synthesis: Results were synthesized using framework previously developed by other researchers such as Pluye and Hong [37] and Noyes [38]. These studies provide a clear way of presenting numerical and narrative data in sequence. The sequence of results were presented, first, in numerical data (mostly in tables) and narrative structure (qualitative data) respectively. Enrollment and dropout status were presented first. Determinants were presented after the findings of numerical data. All results were plotted by their code, converted into simple language and presented in bullets and citation code.
4. Ethical consideration: The research proposal was approved from review board of Policy Research Institute, Nepal. Before starting the FGD, a detailed scope of the study was described to the participants/respondents and written consent was taken in group. The written consent form was adopted from Clemson University South Carolina [39]. It was assured that all data/information provided by the participants would not be disclosed and that they were free to withdraw from the research anytime they wanted.

## Results

### Enrollment status by district in F/Y 2015/16

The national health insurance program was first introduced in 2015 in 3 districts with coverage of less than 1%. Among the three districts, Ilam had the highest coverage (1.42%) while Baglung had the lowest (0.63%) (Table 1).

**Table 1:** Situation of health insurance enrollment in 2015

S. N	District	Total population	Total enrollment	Enrollment %	Subsidy enrollment	Dropout
1	Baglung	277,582	1764	0.63	–	NA
2	Ilam	302,791	4313	1.42	–	NA
3	Kailali	870,771	7468	0.85		NA
	Total	1,451,144	13,545	0.91	**–**	

Source: Health insurance board, Teku, Kathmandu, Nepal

### Situation of enrollment and dropout by districts in F/Y 2016/17

The health insurance program was expanded to twelve more districts during the fiscal year 2016/17. As shown in Table 2, the population coverage increased from less than 1% in 2015/16 to about 5% in 2016/17. Less than 1% of the enrollment was subsidized for marginalized population. However, there was a wide variation in enrollment rates at the districts level with Palpa leading by some margin at an enrollment rate of 17% followed by Kaski (8.02%) and Ilam, (7.26%). At the other end of the distribution was Achham which saw a mere 0.53% of the population enrolled in health insurance during that year. Baitadi, Jajarkot and Kailali—also had dismal rates of enrollment in health insurance program.

**Table 2:** Situation of enrollment and dropout in 2016/17

S. N	Name of the districts	Total population	Total enrollment		Total subsidy enrollment		Dropout	Drop out in %
			No.	%	No.	%	No.	
1	Achham	274,505	1472	0.53	–			
2	Baglung	277,582	16,027	5.77	459	2.86	1035	58.67
3	Baitadi	260,015	3139	1.20	–			
4	Bhaktapur	340,066	13,890	4.08	–			
5	Chitawan	644,219	38,501	5.97	106	0.275		
6	Gorkha	259,299	9107	3.51	253	2.77		
7	Ilam	302,791	21,986	7.26	18	0.08	2240	51.93
8	Jajarkot	186,375	3090	1.65	129	4.17		
9	Jumla	117,958	6763	5.73	714	10.55		
10	Kailali	870,771	13,507	1.55	76	0.56	5846	78.28
11	Kaski	543,767	43,628	8.02	110	0.25		
12	Makawanpur	394,229	17,891	4.53	70	0.39		
13	Myagdi	112,643	6450	5.72	145	2.24		
14	Palpa	255,386	44,355	17.36	0			
15	Tanahu	336,710	9298	2.761	79	0.84		
	Total	5,176,316	249104	4.81	2159	0.86	9121	67.33

Source: Health insurance board, Teku, Kathmandu, Nepal

In the three districts understudy, more than two-third of the population enrolled in health insurance during its inception in the year 2015/16, dropped out of health insurance in 2016/17. The dropout rate was highest in Kailali district where more than 78% of enrollees opted for not renewing their health insurance. This restricted Kailali to an enrollment rate of just about 1.55% much lower compared to other districts that had just adopted the program. Dropout rates in Baglung and Ilam were also alarmingly high.

### Enrollment and dropout status by district F/Y 2017/18

During the fiscal year 2017/18, the government expanded the health insurance program to a total of 37 districts as listed in Table 3. The coverage at the national level almost doubled to more than 9% compared to that of the previous year. Also, the share of the subsidized enrollment skyrocketed from just about 0.89% a year earlier to more than 21%. The district level variation in enrollment rates only widened as district like Palpa achieved coverage of almost 40%.

**Table 3:** Situation of enrollment and dropout in 2017/18

S. N	Name of the districts	Total population	2074/75				Dropout	Drop out in %
			Total enrollment		Total subsidy enrollment (SE)			
			No.	%	No.	%	No.	
1	Achham	274,505	9873	3.59	7719	78.18	1407	95.58
2	Arghakhanchi	200,967	20,973	10.43	6331	30.18		
3	Baglung	277,582	27,444	9.88	8564	31.20	8421	52.54
4	Baitadi	260,015	4003	1.53	5	0.12	2615	83.30
5	Bajhang	210,122	12,190	5.80	9638	79.06		
6	Bajura	146,338	14,497	9.90	11,040	76.15		
7	Bardiya	456,547	63,195	13.84	43,324	68.55		
8	Bhaktapur	340,066	48,730	14.32	21	0.04	4703	33.85
9	Bhojpur	169,139	12,072	7.13	3645	30.19		
10	Chitawan	644,219	138,550	21.50	195	0.140	17,797	46.22
11	Gorkha	259,299	19,860	7.65	3522	17.73	5836	64.08
12	Ilam	302,791	35,903	11.85	14	0.03	10,530	47.89
13	Jajarkot	186,375	20,225	10.85	12,108	59.86	2250	72.81
14	Jhapa	875,828	129,685	14.80	396	0.30		
15	Jumla	117,958	19,602	16.61	11,940	60.91	4584	67.78
16	Kailali	870,771	71,922	8.25	55,207	76.75	10,427	77.19
17	Kalikot	149,371	17,056	11.41	12,696	74.43		
18	Kapilbastu	625,522	14,578	2.33	4888	33.52		
19	Kaski	543,767	75,857	13.95	356	0.46	20,013	45.871
20	Khotang	190,100	13,209	6.94	5837	44.18		
21	Mahottari	673,405	4560	0.67				
22	Makawanpur	394,229	40,653	10.31	130	0.31	7616	42.56
23	Myagdi	112,643	8568	7.60	277	3.23	3395	52.63
24	Palpa	255,386	100,930	39.52	53	0.05	6754	15.22
25	Parsa	663,559	3733	0.56				
26	Pyuthan	236,540	17,470	7.385	4710	26.96		
27	Ramechhap	206,653	9048	4.37	146	1.61		
28	Rautahat	772,098	4333	0.56	35	0.80		
29	Rolpa	232,419	12,208	5.25	6988	57.24		
30	Rukum East	55,903	2811	5.02	1357	48.27		
31	Rukum West	163,968	23,678	14.44	6306	26.63		
32	Sindhuli	305,164	32,852	10.76	19,040	57.95		
34	Solukhumbu	104,415	3401	3.25	5	0.14		
35	Sunsari	845,555	62,737	7.41	146	0.23		
36	Surkhet	387,858	23,587	6.08	23	0.09		
37	Tanahu	336,710	39,406	11.70	9015	22.87	4537	48.79
Total	Total	12,847,787	1,159,477	9.024	245,677	21.18	110,885	44.51

Source: Health insurance board, Teku, Kathmandu, Nepal

The dropout rates at the national level decreased by 22% compared to the year earlier to about 44.5%. However, there was a large variation in dropout rates in the district level with Palpa faring relatively low dropout rate of about 15% compared to Achham where more than 95% of the enrollees dropped out of the health insurance in 2017/18.

### Enrollment and dropout status by district F/Y 2018/19

The health insurance program was expanded to a total of 46 districts during the fiscal year 2018/19 as depicted in Table 4. The coverage of health insurance at the aggregate level increased by two percentage points to reach about 11% during that year while the share of subsidy enrollment increased to about 31% (10 percentage points increase compared to previous year). At the national level, there was also a slight improvement in decreasing dropout rate from 44.5% the year earlier to about 38.4%. However, there was a large variation in both the enrollment as well as the dropout rates in the district level. For example, while Palpa saw more than half of its population covered by health insurance, Mahottari covered only about 1% of its population.

**Table 4:** Situation of enrollment and dropout in 2018/19

S. N	Name of the districts	Total population	2075/76				Dropout	Drop out in %
			Total enrollment		Total subsidy enrollment			
			No.	%	No.	%	No.	
1	Achham	274,505	15,024	5.47	12,177	81.05	7662	77.60
2	Arghakhanchi	200,967	31,212	15.53	13,815	44.26	8652	41.25
3	Baglung	277,582	36,743	13.23	17,174	46.74	9072	33.05
4	Baitadi	260,015	5969	2.29	3658	61.28	2770	69.19
5	Bajhang	210,122	21,098	10.04	18,311	86.79	6465	53.03
6	Bajura	146,338	14,737	10.07	12,669	85.96	9708	66.96
7	Banke	554,630	18,307	3.30	6137	33.52		
8	Bardiya	456,547	57,855	12.67	45,084	77.92	22,311	35.30
9	Bhaktapur	340,066	76,340	22.44	5722	7.49	13,457	27.61
10	Bhojpur	169,139	13,914	8.22	7098	51.01	6276	51.98
11	Chitawan	644,219	156,541	24.29	11,432	7.30	52,506	37.89
12	Darchula	139,712	4685	3.353	1499	31.99		
13	Dhanusa	169,139	4392	2.59	2017	45.92		
14	Dolpa	39,832	858	2.15	116	13.51		
15	Gorkha	259,299	28,880	11.13	11,476	39.73	8752	44.06
16	Humla	55,261	2979	5.39	2247	75.42		
17	Ilam	302,791	49,795	16.44	11,887	23.87	13,985	38.95
18	Jajarkot	186,375	21,161	11.35	13,260	62.66	9917	49.03
19	Jhapa	875,828	191,439	21.85	21,791	11.38	49,856	38.44
20	Jumla	117,958	25,112	21.28	15,135	60.26	6911	35.25
21	Kailali	870,771	98,093	11.26	77,118	78.61	16,230	22.56
22	Kalikot	149,371	17,582	11.77	14,717	83.70	13,230	77.56
23	Kanchanpur	494,553	6165	1.24	1945	31.54		
24	Kapilbastu	625,522	36,870	5.89	22,856	61.99	7036	48.26
25	Kaski	543,767	92,999	17.10	13,559	14.57	25,800	34.01
26	Khotang	190,100	18,055	9.49	14,022	77.66	7917	59.93
27	Mahottari	673,405	7031	1.04	4349	61.85	3704	81.22
28	Makawanpur	394,229	78,891	20.01	3524	4.46	12,102	29.76
29	Mugu	60,109	1555	2.58	549	35.30		
30	Myagdi	112,643	12,287	10.90	2878	23.42	2855	33.32
31	Palpa	255,386	129,782	50.81	7431	5.72	15,475	15.33
32	Parsa	663,559	12,566	1.89	7518	59.82	3062	82.02
33	Pyuthan	236,540	23,826	10.07	14,297	60.0		
34	Ramechhap	206,653	17,184	8.31	7048	41.01	5458	60.32
35	Rautahat	772,098	8988	1.16	3127	34.79	3251	75.02
36	Rolpa	232,419	9566	4.11	6740	70.45	8692	71.19
37	Rukum East	55,903	2439	4.36	1818	74.53	2362	84.02
38	Rukum West	163,968	26,041	15.88	5304	20.36	13,957	58.94
39	Sankhuwasabha	157,854	8304	5.26	2460	29.62		
40	Sindhuli	305,164	39,612	12.98	23,879	60.28	12,681	38.60
41	Siraha	674,923	12,148	1.79	7283	59.95		
42	Solukhumbu	104,415	2361	2.26	1405	59.50	2807	82.53
43	Sunsari	845,555	127,037	15.02	13,523	10.64	23,383	37.27
44	Surkhet	387,858	25,772	6.64	6172	23.94	13,501	57.23
45	Syangja	270,403	34,618	12.80	9130	26.37		
46	Tanahu	336,710	49,692	14.75	17,617	35.45	14,183	35.99
	Total	15,464,203	1,676,505	10.84	522,994	31.19	444,967	38.37

Source: Health Insurance Board, Teku, Kathmandu, Nepal

## Results from qualitative analysis

FGDs explored the characteristics of the households that were not willing to enroll in health insurance program and those who opted to drop out. In addition, during the FGDs, the causes related to service provider (health care facility), program operation by Health Insurance Board and lapses in policy were discussed. The findings of the FGDs are summarized as below.

### Characteristics of participants

Table 5 shows the characteristics of participants involved into focus group discussion. Each district had two focus groups and each group consisted 9–11 participants. More than half of the participants were above 25 years of age. A majority of the participants were female and had secondary level or higher education.

**Table 5:** Characteristics of participants on focus group discussion

Characteristics	Districts					
	Bardiya		Chitwan		Gorkha	
	Group A	Group B	Group A	Group B	Group A	Group B
Age						
< 25	2	2	2	2	3	3
> 25	7	7	9	9	7	7
Sex						
Male	2	1	1	0	1	1
Female	7	8	10	11	9	9
Education						
Up to secondary (SLC)	2	1	1	1	4	4
Above secondary	7	8	10	10	6	6

### Characteristics of unwillingness to enroll and eagerness to dropout

Our FGD with EA first accessed the characteristics of households whowere not willing to enroll and wanted to drop out of health insurance program. It concludes the basic characteristics of the majority of families those were not interested in health insurance and dropped out as reported by EA. These included families of–

1. Teachers,
2. Government employees,
3. Some elected political leaders and relatives,
4. Person in security (Army, Armed Police Force (APF), Nepal Police and pension holders of British and Indian Army because they have their own service hospital and fund to cover during illness for all families,
5. Rich people, businessman, factory/company workers (busy during office hours so they don’t have enough time to go the hospital, frequent travel, need to stay in queue for long hours and receive OPD services which runs during same hours and prefer check-up in private hospitals),
6. Migrant families (those who have gone abroad with their son, daughters, moved to cities for better education and employment opportunities).
7. Ultra-poor people in some districts who didn’t have identification card.

### Factors associated with unwillingness to enroll and keenness to dropout

#### Factors related to health service delivery

(I) Insufficiency of drugs and other essential supplies: There were no sufficient drugs and other supplies particularly for chronic diseases patients due to high utilization of health service and delay of purchasing procedure of drugs and supplies.
(II) Longtime waiting for health service: Health service delivery process was very slow due to high volume of patients, software/server problem, timely  and/or unavailability of health workers, limited doctors, lack of diagnostic facilities etc.
(III) Poor infrastructure and maintenance: People had no attraction with the service of registered health care facilities due to no waiting space, unavailability of safe drinking water, poor waste management, old and unmaintained rest rooms.
(IV) Unfriendly behavior of health workers: Health workers behavior was viewed as a rude one (may be not intentionally), and did not provide adequate and appropriate information. They even disrespected the patients.
(V) Moral hazard of health workers: Discourage to enroll in health insurance by service providers, misbehaving health insurance enrollee, prefer to go to private hospital or pharmacy, company loyalty on drug prescription and discouragement in having access of drugs provided by health insurance. Intentionally, actors in service provider institution’s  prefer private hospitals and clinic to receive the benefit of out of pocket payment by patients.

#### Factors associated with health insurance program implementation

Not only health service delivery, but participants explored that people’s unwillingness to enroll and the desire to drop-out of health insurance if ensured previously was due to many weaknesses of health insurance program implementation. The participants realized that they did not have sufficient knowledge and convincing capacity to encourage the people and health service provider as well. Moreover, there was no timely and effective coordination between different stakeholders about the roll out and the benefits of health insurance program. The participants highlighted that there was a lack of proper coordination among different level of health insurance offices and federal government, local government, health care facilities, political and bureaucratic officials. The gap in communication, collaboration and a situation of silence and conflict appeared as the characteristics of the program implementation. This affected in health service delivery for the enrollee. Ultimately this resulted in drop out from the program. Participants (EA) highlighted that they were not receiving the relevant forms, registers, cards etc. on time so they were not able to enroll more despite their will. Some participants also complained about not getting their incentive on time. Hence, they were not encouraged to intensify the enrollment and to reduce dropout.

#### Factors related to program and policy

The consistency regarding the unit of enrollment in health insurance emerged as a concern. In health insurance act, regulation and ongoing program, family is the unit to enroll in health insurance. But government budget speech provided the subsidy of health insurance for the senior citizen in individual basis while senior citizen were also the member of the family. It created dilemma during enrollment process. The maturation time to activate the policy is 3 months and due to this gap people are not attracted to adopt the HI policy. Health insurance board’s role appeared crucial in developing and practicing different co-payment measures like transportation, choice of hospital, increase the threshold of service etc. which is not incorporated in the scheme at the moment. Due to these policy weaknesses, large proportion of people are not attracted with health insurance program, and those enrolled also are leaving the program.

### Description of representative cases from different districts

#### Bardiya

*One of the female Enrollment Assistants, age 34, from Gulariya participating in a FGD said,* “After a series of visits made by ward representatives, households got enrolled in the program. But they did not renew the policy next year because some of them did not utilize the health service and others were not provided appropriate service. If they had to pay out of pocket for quality services, they felt that there was no need to renew the HI policy”. This suggests that when we talk of high dropout rates, there are multiple factors that contribute to take a decision. In this case there seems a miss match in the expectations of the client and the service providers contributed to drop out. This also indicates that clients were not informed appropriately regarding the philosophy of social security in health care while to participating in health insurance scheme.

*An enrollee expressed his view to the EA (female participant, age*-*26, Gulariya)* “I enrolled in insurance at your request but, I couldn’t receive any services last time and now I don’t think it’s perfect for me as I have to go to the tertiary care hospitals either in Kathmandu or Luknow for specialized treatment; I don’t want to renew this time”.

*Participant from Saurah primary hospital Bardiya* said that X-ray machine was provided in Sauraha primary hospital 2 years ago but still it is not in use. We had talked with ward chairman of PHC about this issue, but he said he doesn’t know about it. Gaupalika (local government body) chairman said “We shall do something to operate it” however, it is not used yet. So it’s almost certain that the equipment will no longer be usable. At this instance, there remains an operational issue at the ground when it comes to implementation of health insurance scheme in the districts. This further points out to the fact pertaining to weak management of the program and inappropriate communication that existed.

#### Chitwan

*Another female participant of age 28, from Mangalpur said,* “I took my son to the hospital after he got a fracture. First I took him to Bharatpur hospital but they didn’t examine the case in detail. Instead they said that they had ortho campaign going on at the moment and hence it would take about 1 week to look after this case. They didn’t even do the X-ray. Then I took my son to Chitwan Medical College (CMC) which is listed as health care provider by health insurance board, got surgery done and received full care. I received service on our own effort after series of consultation and discussion”. At this instance it is about negligence. Now what led to such negligence must be explored while to discuss the high dropout rates. Given the data from the field, it is clear that it was not only enrollment and the services allowed under the coverage package but was also about the priority, attitude and the education that the health worker had on implementation of national health insurance. This case clearly suggests that at this instance, it was about cost–benefit analysis done by the service provider that resulted in a particular attitude while to provide services.

*One female participant of age 34, from Bharatpur,* directly observed in hospital that during checkup, doctors de-motivated (don’t renew health insurance, there is not good medicine available).

#### Gorkha

*One female EA of age 38, from Borlang* shared her experience**—**‘Her ward secretary took his daughter to the hospital for a surgery. The operation was not done on time, and hence EA told, “Don’t do this health insurance, it doesn’t work well, and this is only to exploit people”. Thus it appears that even enrollment assistants were not convinced of the scheme under operation.

*Another male EA of age 26, from Palungtar* shared his experience from his ward chairperson *regarding enrollment and renew of health insurance. He argued that* “Health Insurance is not compulsory. We don’t know whether services will be available or not. Many people complain about this and  did get enrolled just for the sake of it”. Some even said that they will enroll they reach 70 years of age.

## Discussion

Based on both qualitative and quantitative data drawn from the field in three districts of Nepal, health insurance coverage in regard to enrollment and dropouts are presented with appropriate analysis. In Nepal, health insurance program was rolled out in phases. First the national health insurance scheme was introduced in 3 districts. It was then extended to 15 districts in the second year and to 35 districts until 2018. In 2019, HI was extended to 46 districts. The enrollment coverage is only 11% (2019) as covered districts in total population, the subsidy enrollment (for the vulnerable population) is increasing (< 1%, 21% and 31%). Dropout rate is decreasing but it is not solely due to increasing enrollment in total population but due to increasing subsidy enrollment and financially they need not to pay the premium. Widening the subsidy groups and free enrollment in health insurance program by state is however a challenge for program sustainability as large portion of government revenue is consumed [40]. Dropout of health insurance is high in plain (Tarai) districts. The demographic characteristics of health insuree were, government employees, local political leaders, high class families, businessman. There were three types of factors responsible for enrollment and dropout of health insurance, namely demographic, program and policy related issues and means of health service delivery including the intention of health workers.

There is no threshold regarding the enrollment and dropout rate in different countries. In low and middle income counties like Nepal, there is poor enrollment and high dropout rate. Our study shows that dropout rate was 67%, 44% and 38% in consecutive 3 years between 2016 and 2018. A study in Nigeria shows that there is poor enrollment and dropout rate is 4–52% by different scheme [41]. Another study in Ghana explored that dropout rate had increased from 41 to 53% within 1 year period [41]. A study by Mebratie et al. found that dropout rate was 25% in Ethiopia [42]. The above studies present similar findings as this study indicates. Further, an unwillingness to get enrolled persists particularly from the sections of government employee, business and well to do families, migrant population, and those who did not become sick last year and the ultra-poor. This shows similar result when compared to other studies. For instance, a study conducted by Boaeting in Ghana showed that male public sector employees reflected reluctance twice than general insuree when it came to having willingness to  get enrolled in HI and opt for dropout [43]. These results are similar with the findings that have emerged from this study. Another study in Ghana by Atinga revealed that those who could not afford the premium were not able to enroll and attributed for dropout [44]. A study by Mhing in Vietnam found that rich and middle income families had high dropout than low income family [45]. All these findings are in congruence with the findings that have emerged from this study.

From the study result, it is clear that the major determinants for enrollment and high dropout is due to unfriendly behavior of health workers and unavailability of drugs in health care facilities. Usually, clinicians discourage the members of health insurance and give more priority to the general patients who could be beneficiaries for out of pocket payment. A qualitative study in Ghana by Debpuur concluded that health workers preferred private clinic for the investigation and treatment and hence ignored the health insurance members [46]. A study by Barati conducted in Iran found that the behavior of health workers varied susbstantially compared to insured and noninsured patients. During the treatment health workers indulged in rude behaviors to the insured patients [47]. On the other hand, there is high utilization of health service and insufficiency of drugs and insured patients could not get adequate drugs from the hospitals. Another study in Saudi Arabia concluded that there is no rational in prescribing of drugs to the insured and uninsured patients in terms of company and variety of drugs [48]. There is a practice of prescribing medicine from a particular company based on the individual benefit of  the physician [49, 50] and participants of this study also reported such behaviors contributing to high drop out.

There are some reasons for poor enrollment and dropout due to program and policy related factors as well. There is no choice available for consumer to take health insurance with copayment. Some high-class households could take those services with more copayment. A systematic review by World Health Organization (WHO) showed that after the copayment policy in China, Taiwan and South Korea, the coverage increased and dropout was reduced [51]. Further, in Nepal, the  current policy is designed in such a way that it does not cover health risks immediately after enrollment. Insuree would get the health service if they renew the policy before 3 months of expiry date or after renewing the policy. Health service will be provided only after 3 months. It means, there is no risk coverage for the gap period of 3 months. Likewise, EA could not properly educate people about the importance of social health insurance.  Also people might have a perception that there is no difference between general (life and materials) insurance and health insurance. As a result, people want to enroll or renew health insurance when they get sick. Even, data indicated that there was no proper coordination between health insurance branches, provincial and local government, hospitals and civil society. For the wide coverage of health insurance, there is need of coordination and trust among different stakeholders [52, 53]. Hence, high coverage of health insurance with nominal dropout remains a challenge. It has been furthermore problematic due to existing mind set of the people, politics and governance at play [27, 54]. In otherwords, it is cumulative challenge of people's awareness, available policies and political direction. 

## Conclusion

Success of social health insurance depends on the philosophical direction, theoretical models it adheres to implement the scheme and the high moral and ethics in which schemes are implemented. Nepal has adopted the hybrid model of social health insurance in terms of financial (low cost), service (limited service) and population coverage (lower middle income group). However there is a need to fix the model while drawing upon the philosophy of socialist democracy as envisioned in the constitution of Nepal 2015. It needs to revisit existing HI policy, structure (Infra structure and human resource) and service delivery mechanism. During program operation as purchaser, health insurance board needs to properly address the subsidy enrollment. They need to include a wide range of health insurance packages, and have a proper monitoring mechanism of health care and health workers, easy enrollment system, and a couple of alternative ways to pay the premium. Considering the geographical situation of Nepal, the referral period from one health facility to another is just 7 days and need more time. Some of the issues that are discussed are vital for sustainability and effective implementation of health insurance in Nepal. It is clear that there is an imbalance in terms of people’s expectations to health insurance and the institutions who cater the service. Also, health worker’s complicit attitude against health insurance plays an important role in implementation of the scheme. Hence HI in Nepal needs obligatory enrollment to all citizen. The Implementation of the scheme needs to be extended in full fledged manner to (not phase wise) all over the country. Varieties copayments modalities need to be introduced in effective coordination with different actors. To further reach out to the people in the margin awareness program to the people and capacity building of EA need to be strengthened. Further researches would be fruitful to explore the capacity of health care facilities to adopt health insurance and people’s demand on different type of health services.

### Notes and study limitation

This study was conducted through Policy Research Institute, Kathmandu as per the request of the Health Insurance Board (HIB). There was time limit to explore all factors responsible for drop-out of health insurance while representing all region, province and representative districts. The findings from qualitative study have its limitation and hence may not represent all districts, province and country. Moreover, there is a possibility of information and recall bias from the respondents.

## Data Availability

Not applicable.

## Abbreviations

APF: Army, Armed Police Force
CBHI: Community Based Health Insurance
EA: Enrollment Assistant
HIB: Health Insurance Board
OPP: Out of pocket payment
PI: Principal investigator
PRI: Policy Research Institute
SDG: Sustainable Development Goal
SPH: Social protection of health
UHC: Universal health coverage
UN: United Nations
WHO: World Health Organization
